# A unique presentation of *NLRP3*-associated autoinflammatory disease: case report

**DOI:** 10.1186/s41927-022-00321-8

**Published:** 2022-12-12

**Authors:** Stéphanie Ducharme-Bénard, Guillaume Roberge, Hugo Chapdelaine

**Affiliations:** 1 Department of General Internal Medicine, Hôpital du Sacré-Coeur de Montréal, Université de Montréal, 5400 Boulevard Gouin Ouest, Montreal, H4J 1C5 QC Canada; 2 Centre d’excellence en maladies vasculaires, Hôpital St-François d’Assise, Centre Hospitalier Universitaire de Québec–Université Laval, 10 rue de l’Espinay, Quebec, QC G1L 3L5 Canada; 3 Department of Allergy and Immunology, Centre Hospitalier Universitaire de Montréal, Montreal, QC Canada; 4 Institut de Recherches Cliniques de Montréal, 110, Avenue des Pins Ouest, Montreal, QC H2W 1R7 Canada

**Keywords:** *NLRP3*, Autoinflammatory disease, Cryopyrin-associated periodic syndrome, CAPS, NOMID, CINCA, Eosinophilia, Serositis, Arthritis, Case report

## Abstract

**Background:**

*NLRP3*-associated autoinflammatory diseases (*NLRP3*-AID) are rare genetic autoinflammatory diseases characterized by chronic inflammation and an urticaria-like rash. We report an unusual presentation of severe *NLRP3*-AID resulting in a significant diagnostic delay of more than three decades.

**Case presentation:**

The patient presented with early-onset serositis as well as prominent peripheral eosinophilia with organ infiltration, in the absence of the classic urticaria-like rash. DNA analysis by next generation sequencing revealed a sporadic class 4 mutation c.1991T > C (p.Met662Thr) in the *NLRP3* gene, confirming a diagnosis of *NLRP3*-AID at 36 years old. Although treatment with anti-interleukin 1 agent led to clinical remission, irreversible sequelae, namely intellectual disability and deafness, remained.

**Conclusion:**

This case highlights unique manifestations of *NLRP3*-AID, namely the absence of urticaria-like rash, eosinophilic organ infiltration, and pseudoseptic serositis. In order to avoid diagnostic delay and its dire consequences, *NLRP3*-AID should be suspected in patients displaying autoinflammatory features combined with serum and tissue eosinophilia and/or marked serositis, regardless of skin involvement.

## Background

*NLRP3*-associated autoinflammatory diseases (*NLRP3*-AID), formerly named Cryopyrin-associated autoinflammatory syndrome (CAPS), are rare genetic autoinflammatory diseases associated with gain-of-function mutations in *NLRP3* coding for cryopyrin [1]. They can present with a mild, intermediate, or severe phenotype. As embodied by recently published classification criteria, cardinal manifestations involve chronic inflammation associated with cold-induced symptoms, an urticaria-like rash, arthralgia and/or arthritis, sensorineural hearing loss, chronic aseptic meningitis, and skeletal deformities [2]. We hereby report a severe case of *NLRP3*-AID presenting with unusual manifestations, namely eosinophilic organ infiltration and pseudoseptic serositis in the absence of skin involvement.

## Case presentation

A 36-year-old French Canadian male presented with a 3-week history of left knee arthritis. Blood work revealed leukocytosis with predominance of neutrophils at 23 × 10^9^/L, microcytic anemia with hemoglobin at 95 g/L, and markedly elevated C-reactive protein (CRP) at 321 mg/L. Left knee radiograph revealed significant joint effusion. Synovial fluid analysis was abnormal with 161 500 white blood cells/ml (91% neutrophils) and low glucose level at 0.8 mmol/L, consistent with septic arthritis. The patient was treated accordingly, with no clinical or biochemical improvement. Extensive microbiological investigations were negative. An underlying inflammatory disorder was therefore suspected. High dose oral prednisone was started, leading to marked clinical improvement and decrease in CRP from 271 to 106 mg/L within forty-eight hours. Work-up for autoimmune diseases revealed positive HLA-B27 antigen and rheumatoid factor at 160 IU/mL; antinuclear antibodies, extractable nuclear antibodies, anti-double stranded DNA, antineutrophil cytoplasmic antibodies and anti-cyclic citrullinated peptides were negative, while complement levels were normal.

One week later, the patient complained of chest pain, with concomitant increase in inflammatory markers. Imaging revealed a right loculated pleural effusion. Fluid analysis revealed the presence of a non-purulent exudate with markedly low pH, undetectable glucose level, and negative gram stain. Although pleural white blood cells were increased at 7300/uL, differential count was not available. Antibiotics were resumed for suspected complicated parapneumonic effusion. The dose of prednisone was halved to 25 mg daily. Chest tube drainage was unsuccessful. Pleural thrombolysis was therefore attempted and led to massive hemothorax with respiratory distress requiring urgent thoracotomy and decortication. Pleural bacterial, fungal, and mycobacterial cultures were negative. A 4-week course of antibiotics was administered, while moderate dose prednisone was maintained. Upon discharge, knee synovitis and pleural effusion had resolved, but CRP remained elevated at 111 mg/L.

Given the uncertainty of the underlying inflammatory disorder, immunology was consulted. Family history was found to be unremarkable, with no consanguinity. Personal history revealed first admission at 2 years old for aseptic meningitis. Sensorineural hearing loss, macrocephaly, and global developmental delay were concurrently diagnosed in the setting of elevated inflammatory markers, namely leukocytosis with eosinophilia. The latter was persistently superior to 1500/mm^3^, despite the absence of atopic manifestations including urticaria, negative allergy tests, and normal immunoglobulin E level. A maculopapular rash was, however, noted on the patient’s abdomen on a few occasions between 7 and 8 years, with no recurrence afterwards. Cerebral CT scan revealed diffuse atrophy. A few years later, recurrent episodes of hyperthermia and lower extremity arthralgia led to a diagnosis of juvenile idiopathic arthritis. Aspirin was administered, with transient improvement in symptoms. In light of persistent inflammation and failure to thrive, upper and lower gastrointestinal endoscopies were performed revealing eosinophilic enterocolitis. Prednisone was given for several months, which partially reversed growth retardation in spite of persistent elevation in inflammatory markers. Pericarditis, with hemodynamically significant pericardial effusion requiring drainage, as well as unilateral pleural effusion, left elbow arthritis, and fever developed at 6 years old. Microbiologic work-up was negative. Aspirin was resumed, with improvement of symptoms.

**Fig. 1:**
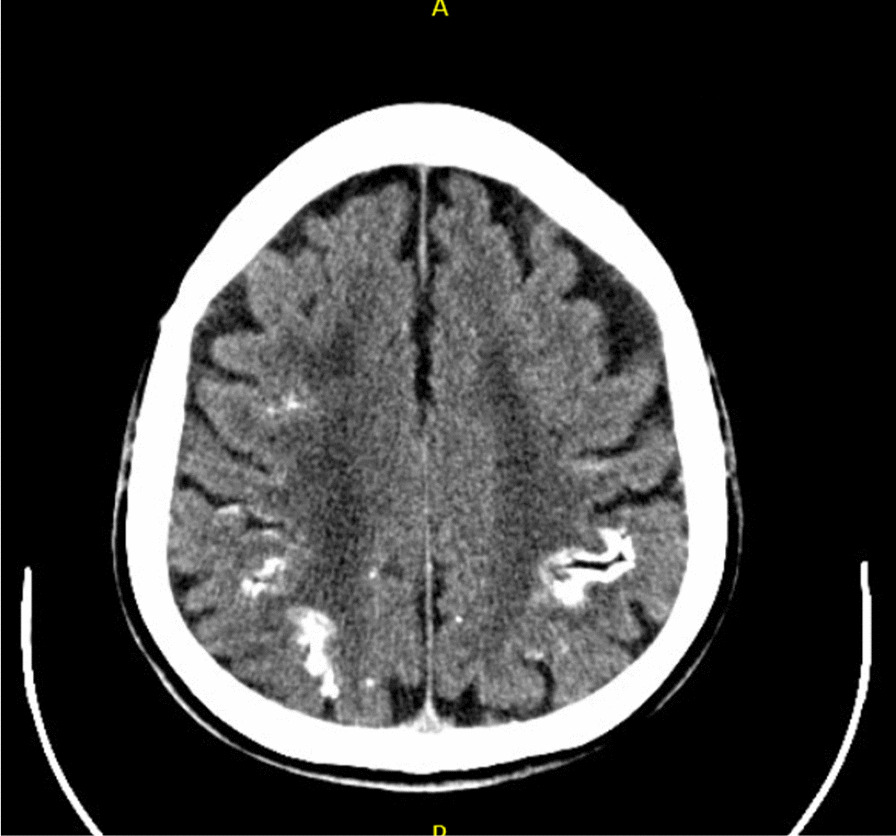
Head CT scan showing diffuse brain atrophy with cortical calcifications

During the following years, global developmental delay persisted. Pseudopapilledema was diagnosed during adolescence. Repeat cerebral CT scan revealed diffuse brain atrophy with cortical calcifications (Fig. 1). The patient developed alternating buttock pain relieved by non-steroid anti-inflammatory drugs (NSAIDS). Radiographs showed sclerosis and possible erosion of the right and left sacroiliac joints, respectively. In light of positive HLA-B27 status, spondyloarthritis was diagnosed and treated with NSAIDS as needed. During adulthood, the patient continued to suffer from intermittent arthralgia. He was treated twice for suspected septic arthritis of the right wrist, despite negative microbiologic work-up and absence of crystals on joint aspiration.

**Fig. 2:**
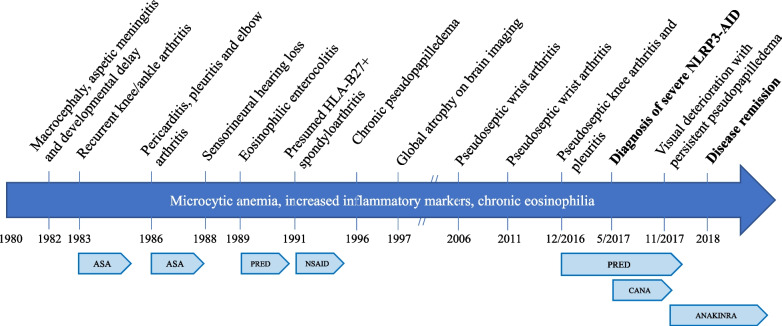
Overview of clinical evolution. Abbreviations: ASA: aspirin, PRED: prednisone, NSAID: non-steroidal anti-inflammatory drug, CANA: canakinumab

In light of the long-standing history of sensorineural hearing loss, physical and mental developmental delay, intermittent serositis, and elevated inflammatory markers, *NLRP3*-AID was suspected. DNA analysis by next-generation sequencing of 36 genes implicated in autoinflammatory diseases was normal, except for a heterozygous class 4 mutation in exon 3 of the *NLRP3* gene, c.1991T > C (p.Met662Thr) [3]. No *NLRP3* mutation was detected in the patient’s parents. The mutation had previously been described in two cases of severe *NLRP3*-AID [4,5]. Canakinumab was started at a dose of 150 mg every 2 months, then increased to 300 mg every month. Prednisone was initially maintained at 20 mg daily, then tapered over a few months. Although inflammatory markers almost normalized, visual deterioration prompted a switch to anakinra 100 mg daily after 6 months. The latter allowed clinical stabilisation with resolution of pseudopapilledema within a few months. Unfortunately, intellectual disability and deafness persisted. He has now been treated with anakinra for over 4 years, with no disease recurrence. Clinical evolution is summarized in Fig. 2.

## Discussion and conclusions

The proband displayed unique manifestations of severe *NLRP3*-AID, namely the absence of urticaria-like rash, peripheral eosinophilia with organ infiltration, and prominent serositis. These manifestations were not observed in the two previous cases bearing the same mutation, highlighting the absence of a clear genotype-phenotype correlation in this disease.

Skin rash is thought to be a cardinal feature of *NLRP3*-AID. It can migrate and fluctuate in intensity but is usually persistent or recurrent throughout life. According to the EUROFEVER series, an urticaria-like rash is present in 89% of patients; only 3% lack cutaneous manifestations [6]. Although our patient did have a rash on the abdomen a few times, its non-urticarial and non-persistent character is quite atypical for *NLRP3*-AID, raising the possibility that it was unrelated to the latter.

Eosinophilia has been reported in moderate to severe forms of *NLRP3*-AID [7,8,9] In a retrospective study, serum eosinophilia above 1500/mm^3^ was reported in 7.9% of the 76 *NLRP3*-AID patients involved [10]. However, eosinophilic organ involvement in the setting of *NLRP3*-AID appears rarer. In the first case series of severe *NRLP3-*AID by Prieur et al. in 1987, eosinophilic infiltration of the skin, lymph nodes, and cerebrospinal fluid was described [8]. In 2001, liver biopsy revealed the presence of an inflammatory eosinophilic infiltrate in a patient with severe *NLRP3*-AID [9]. Our patient developed eosinophilic intestinal infiltration, which has never been reported. The pathogenesis underlying serum and tissue eosinophilia in *NLRP3*-AID remains unclear. NLRP3-deficient mice were shown to have reduced serum interleukin-5 level and lung eosinophilia, suggesting that the NLRP3 inflammasome induces production of pro-eosinophilic cytokines [11]. Indeed, functional analysis of peripheral blood mononuclear cells in *NLRP3*-AID patients revealed increased secretion of interleukin-3 and − 5, which both stimulate eosinophil production [7].

Although less prevalent than in familial Mediterranean fever and Tumor necrosis factor receptor-1 associated periodic syndrome, inflammation of pericardial and pleural membranes has been reported in *NLRP3*-AID [12,13,14]. Our patient presented two distinct episodes of pleuritis, and one episode of pericarditis. He also developed three episodes of pseudoseptic arthritis. Each of these episodes had no clear trigger, lasted a few weeks up to 2 months, and recurred every few years. Although inflammatory arthritis and more rarely erosive synovitis are well documented in the spectrum of *NLRP3*-AID, only one case report has reported results of synovial fluid analysis performed in two patients [15]. The latter revealed markedly elevated white blood cell count similar to our patient.

In summary, we report a unique case of severe *NLRP3*-AID associated with a sporadic *NLRP3* mutation c.1991T > C who presented with serum and tissue eosinophilia, pseudoseptic serositis and no urticaria-like rash, leading to marked diagnostic delay and irreversible sequelae. Therefore, *NLRP3*-AID should be suspected in patients displaying autoinflammatory features combined with serum and tissue eosinophilia and/or marked serositis, regardless of skin involvement.

## Data Availability

The genetic data presented in the current study is available in the Infevers repository, [https://infevers.umai-montpellier.fr/web/detail_mutation.php?Id_ mutation = 127]”.

## Abbreviations

*NLRP3*: *NLRP3*-associated autoinflammatory diseases
CAPS: Cryopyrin-associated autoinflammatory syndrome
CRP: C-reactive protein
NSAIDS: Non-steroid anti-inflammatory drugs
